# Pharmacy Undergraduate Education: Can Student Primary Care Placements Add Value to Learning and Teaching?

**DOI:** 10.3390/clinpract16010005

**Published:** 2025-12-25

**Authors:** Amit Bharkhada, Neena Lakhani, Sandra Hall, Martin Grootveld

**Affiliations:** 1 Leicester School of Pharmacy, De Montfort University (DMU), The Gateway, Leicester LE1 9BH, UK; smhall2155gmail.com (S.H.); mgrootveld@dmu.ac.uk (M.G.); 2 Leicester, Leicestershire, Rutland (LLR) Training Hub, Leicester LE7 2EQ, UK

**Keywords:** general practice, primary health care, polypharmacy, medicines optimisation, pharmacy education, patient safety

## Abstract

**Background:** Clinical pharmacists are increasingly demonstrating their value within primary care; this process directly improves patient experiences and outcomes. However, many undergraduate pharmacy students have little or no experience in this area, so that when they first qualify from training, their previous lack of exposure could affect future patient care in this environment. **Aim:** This study aims to evaluate how pharmacy undergraduate students’ learning and development of competencies are managed and received by general practitioner (GP) educators, clinical tutors, students, and patients in a general practice setting. **Design and setting:** The General Practice Pharmacy Educational Placement (GPEP) for undergraduates was designed and delivered in half-a-day each week across five weeks in general practice. Students observed patient consultations, interviewed patients, conducted medication reviews, used medicines reconciliation techniques, and also produced patient care plans. **Method:** Students participating in GPEP completed both pre- and post-course questionnaires rating eight learning outcomes, using a five-point Likert scale. Data analysis incorporated multivariate principal component analysis (PCA) and partial least squares-discriminant analysis (PLS-DA) strategies, and thematic analysis was applied to student focus groups, patient interviews, and GP staff interviews. Following the patient medication interview, students recorded findings and potential interventions for consideration. **Results:** A total of 112/157 students (71%) completed the questionnaires (October 2017–March 2019), with significant statistical differences in student confidence (*p* < 0.0005 for all learning outcomes). Thematic analysis revealed largely positive attitudes towards GPEP. Healthcare professionals highlighted benefits and challenges of GPEP. More than 40 issues relating to medicines optimisation and patient safety were identified, with some requiring immediate interventions from GP teams. **Conclusions:** GPEP demonstrated a positive clinical impact, improving patient safety. The undergraduate activities may encourage students to consider roles in primary and community care, enabling a resilient and able future workforce.

## 1. Introduction

Although general practice in the NHS provides universal access in delivering patient care [1], demand for appointments, high workloads, and staff shortages renders working therein somewhat challenging [2]. To mitigate this and to recognise unmet GP target numbers, a strategy to invest in and recruit allied healthcare professionals, focussed on and supported by the principles guided in the NHS Long Term Plan, was established [3].

This has enhanced opportunities for clinical pharmacists to be recruited into primary care, though their existence in this setting is not entirely new. In fact, this was introduced in Scotland in the 1990s. In 2015, NHS England/Improvement co-ordinated a pilot study, which subsequently led to funded pharmacists being employed in general practice [4]. These evaluations were published and have contributed to NHS commissioning plans for primary care [5,6].

A qualitative review by Ryan et al. [7] examined stakeholders’ views relating to the inclusion of pharmacists in general practice and identified that once communication processes and inter-professional trust are established, decreased workloads and increased patient safety are observed.

However, for these pharmacy roles to be successful, GP practices need to attract a wide range of hopefully experienced applicants. However, many pharmacy undergraduates and newly qualified pharmacists have little or no experience of primary care operations and therefore may be reluctant to apply for these roles as part of the future NHS transformation plans.

United Kingdom (UK) pharmacy undergraduate programmes currently focus on expertise in pharmacology and therapeutics of medicines. However, this undergraduate training lacks significant patient contact, and hence provides only limited opportunities for the development of clinical and communication skills during training, a key area featured in primary care.

Recognising this to be important, in 2015, De Montfort University (DMU) and the Leicester, Leicestershire, and Rutland (LLR) Training Hub partnered and collaboratively developed a General Practice Pharmacy Educational Programme (GPEP) for undergraduates, offering them the opportunity to explore and understand possible future roles within primary care [8,9].

## 2. Study Aims

The broad aims of this retrospective study were to evaluate and understand the impact on how the learning in general practice is managed and received by all stakeholders, specifically (1) the primary healthcare team (PHCT); (2) the clinical tutors; (3) patients interviewed by the students; and (4) students developing their competence in this area.

## 3. Methodology

### 3.1. Study Design

GPEP was designed to enhance undergraduate pharmacy training in a primary care setting (across three GP sites) and determine if transformational learning arose from the interactional discourses between the students, PHCT members, and patients [10].

All pharmacy students had signed a code document before commencing GPEP. They observed patient consultations, interviewed patients, conducted medication reviews, used medicines reconciliation techniques, and developed patient pharmaceutical care plans for their interviewed patients. They shared their different experiences with facilitators (GP trainers, practice pharmacists, and academic DMU staff), who explored learning concepts through debate, discussion, and reflection [11], each sharing patient safety considerations.

GPEP was delivered over two academic years comprising 2.5 days over a 5-week period for 6 cohorts (8–12 students per cohort) per academic year. Over 40 patients were involved in this programme, and all were fully consented by the GP practice training sites. Table 1 provides the framework of the educational programme.

### 3.2. Study Research Ethics Approval and Analysis

The study was defined as an “Evaluation” by the NHS Research Ethics Committee (REC), and full ethical approval was provided by DMU’s Faculty of Health and Life Sciences Research Ethics Committee (approval reference number 3110). Written informed consent was obtained from all participants involved in the study outlined. No participants recruited to the study provided any identifying information.

In order to clarify the “Evaluation” term further, research aims to produce new, generalisable knowledge and test hypotheses, whereas evaluation assesses the effectiveness of one or more specific programmes designed to provide improvements [12].

The study was investigated under three phases: Phase 1: Quantitative studies; Phase 2: Qualitative studies; and Phase 3: Impact and value. For Phase 1, the methodology used is based on the KOLB Cycle [13] and involved pre- and post-intervention Likert scale surveys—a validated and widely accepted approach in UK higher education for evaluating modifications/upgrades in student learning outcomes. It is strongly supported by guidance from Transforming Access and Student Outcomes (TASO, an independent hub for the higher education sector), the Education Endowment Foundation (EEF), and leading UK universities. This approach was also endorsed and validated by internal staff Programme Management Committees, which oversee this and other educational programmes such as Interprofessional education (IPE), etc. The methodology of this research proposal, including the questionnaires featured, were also critically reviewed, approved, and validated by DMU’s Faculty of Health and Life Sciences Research Ethics Committee (FREC). Moreover, the strategy employed readily aligns with sector-wide best practices for evaluating intermediate outcomes for student learning such as knowledge, skills, and attitudes [14].

Internally-validated questions were selected based on learning outcomes designed by the module team leads—these had formed part of previous evaluations. Overall, the study was considered to have a retrospective experimental design.

#### Study Inclusion Criteria

Patients were identified using purposeful sampling by the GPs. They were all adults (>18 year.). The GPs were aware of the GPEP programme and looked at what patients were consulting them on the day and then identified suitable patients with multiple co-morbidities and/or multiple medications that could be suitable for a supervised interview with pharmacy students. Some GP practices also invited patients who consented to be interviewed at a time suitable for both them and the students conducting the interview. It was a ‘random’ selection and also relied on convenience from a practice as well as a patient perspective.

### 3.3. Phase 1: Quantitative Studies

An undergraduate pharmacy student questionnaire was designed in three sections.

In Section 1, students completed the questionnaire before GPEP commenced (pre-learning). Following completion (post-learning), students completed Section 2, where the questions remained the same so that pre- and post-programme scores could be compared, relating to their confidence across eight learning objectives against a 5-point Likert scale. Notably, 157 students initially responded to the pre-questionnaire; however, we were only able to match 112 students who completed both pre- and post-event questionnaires.

In Section 3, which ascertained students’ views on GPEP, there were five closed questions on a Likert scale (analysis from Section 4.1 below), along with five open-ended questions (analysis in Section 4.4 below). A copy of this student questionnaire is available in the Supplementary Information Section S1.

#### Statistical Analysis of Experimental Likert Scale Data

For the experimental Likert scale dataset acquired, differences between the pre- and post-programme scores were analysed using a non-parametric paired Wilcoxon signed rank test (*MetaboAnalyst 5.0*), with the minimum significance level being set at 0.05/8 = 6.25 × 10^−3^ to correct for false discovery rates (FDRs) arising from the testing of eight question variables (Bonferroni correction). Multivariate (MV) principal component analysis (PCA) and partial least-squares discriminatory analysis (PLS-DA) of the before vs. after dataset was also conducted with *MetaboAnalyst 5.0*. The dataset was analysed raw, i.e., the normalisation, transformation, and autoscaling methods were not applied prior to analysis. Since ordinal Likert scale data are not normally distributed, MV statistical analysis was based on Spearman’s rank rather than Pearson correlations. The analysis of ordinal (Likert) scale variable datasets by MV analysis techniques such as PCA has been previously described by Jörekog [15] and Kolenikov and Angeles [16]. Moreover, the use of ranked, non-parametric correlation coefficients such as that of Spearman for MV PCA have been recently described and validated by Asaliontin et al. [17]. Indeed, this research has strongly indicated that such non-Pearson correlations effectively sustain the non-linear correlation structural components often existing between ‘predictor’ variables, and is more effectual computationally, most notably for structural equation modelling protocols. Hence, it can serve to surmount the limitations of this technique commonly experienced when Pearson correlation coefficients are used for the correlation matrix instead [18]. Score plots of PC2 and PC3 vs. PC1 are shown in Figure S2.1 (Supplementary Materials Section S2) for our experimental Likert scale experimental data, where PCA was conducted with either Polychoric, Spearman’s Rank, or Pearson correlation coefficients in the model correlation matrices. Although the results acquired are quite similar in the context of group clustering distinction capacities, it certainly appears that the use of Spearman’s Rank correlation coefficient yielded improved results regarding PC2, with a larger difference between this PC’s group centroids (i.e., −0.16 and +0.16) than those achieved with PC1 or PC3.

Out of 157 students initially recruited, 112 successfully completed Section 1 and Section 2 of the programme, i.e., none of them failed to complete any of the Likert scale responses to all eight questions put to them, both pre- and post-event.

Likewise, MV analysis was also conducted using the empirical Bayes analysis (EBA, otherwise abbreviated as EBAM for the analysis of microarray systems). The approach employed was non-parametric for the analysis of paired Likert scale datasets (after vs. before programme training), a delta value of 0.90 (posterior probability between 0 and 1 specifying differential expression), with the intra-participant group variances being set as equivalent, which indeed they were. The cut-off threshold limit was 0.946.

Contingency table analysis, together with Fisher’s exact test of its cellular contributions, was performed using *XLSTAT2021* software (Addinsoft, Paris, France).

### 3.4. Phase 2: Qualitative Studies

Qualitative data from PHCT members, patients, and students was collected between August 2018 and July 2019.

One-to-one, in-person interviews were conducted with six PHCT members for 20–40 min. (each was provided with a participant information sheet), recruited by the practice manager. Informed consent to record with audio and/or video tapes was obtained. Questionnaires (open-ended and closed questions) were also distributed for PHCT to complete.

Patients taking part in GPEP were contacted by the practice manager to participate in one-to-one interviews. Five adult patients (>18 years old) consented to participate. Two consented to audio recording, and three declined, and so field notes were written to represent their views. Each interview was of 30–45 min. duration. In addition, one patient from the patient participation group (PPG) chose and consented to share their view of the programme via email.

Student views were captured by invitation to a representative focus group. An information leaflet was provided. Student groups were purposively sampled to represent the sites that participated in GPEP, proportionate to those that had the greatest number of students. Two focus groups were convened at two of the training sites (n = 18). All students consented to being digitally recorded, and later content was transcribed ad verbatim by an independent transcriber. Field notes were also taken by the moderators and used in the analysis. The semi-structured interviews, through the use of the topic guide, lasted between 60–90 min.

### 3.5. Phase 3: Impact and Value

In their discussions with the patient, the students produced a pharmaceutical care plan that formed part of their learning objective. The study team analysed the content of these presentations and the need for potential interventions that might relate to medicines optimisation or patient safety that the PHCT members would need to act on. If identified, these would be communicated and escalated to appointed GP site leads for action.

## 4. Results

### 4.1. Phase 1: Quantitative Studies

Between June 2016 and March 2019, 157 pharmacy students completed GPEP. Questionnaire datasets obtained from 112 students, who completed Section 1 and Section 2 of the programme, were analysed (response rate 71%).

Table 2 shows the GPEP learning objectives. Quantitative analysis of the pre- and post-programme data on a paired basis revealed statistically significant differences between student confidences for all the learning objectives featured. The most highly significant difference was found for Q3, which requested information on student understanding of the rationale underpinning medicines optimisation, as might be expected, and the highest mean improvement levels found were for Q5 and Q8.

### 4.2. PCA, PLS-DA and EBAM Analyses

**Figure 1:**
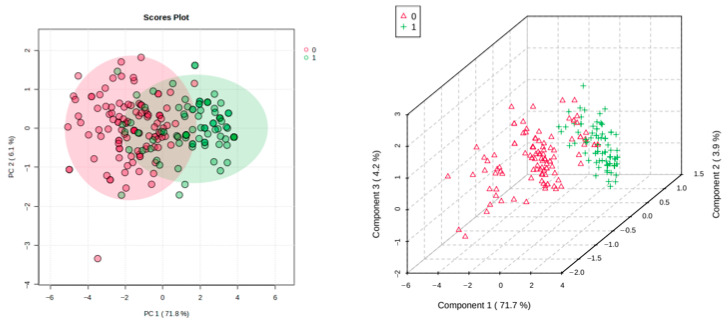
(**Left**): 2D PCA score plot of PC2 vs. PC1. (**Right**): PLS-DA 3D score plot of PC3 vs. PC2 vs. PC1 for the Likert scale data acquired. Key: red triangles classified as 0, post-programme; green crosses classified as 1, pre-programme.

Primarily, both PCA and PLS-DA techniques were applied to the untransformed and non-autoscaled ‘raw’ Likert questionnaire dataset, and these strategies showed clear distinctions between the students’ questionnaire responses completed pre- and post-programme training, with a range of correlated positive responses for the assessment questions completed. Corresponding 2D and 3D score plots are shown in Figure 1. PCA yielded a PERMANOVA R^2^ value of 0.52 (*p* = 0.001 based on 999 permutations), whereas PLS-DA gave a cross-validation Q^2^ value of 0.635 for a model with three components (permutation test *p* value < 5.0 × 10^−4^).

This PCA approach revealed that all questions (Q1–Q8) loaded significantly on PC1, although questions Q4 and Q6 were found to negatively and positively load, respectively, on PC2 too (Q2 and Q8 had the lowest and highest contributions, respectively, towards PC1). However, it should be noted that the low percentage contribution of PC2 to the total variance (6.1%) is partially accounted for by the dual loadings of Q4 and Q6 on both these PCs.

With this analysis, it appears from PC1 PCA loadings that all question responses have the same objectives or sources, as indeed they do, although the significant PC2 loadings of Q4 and Q6 indicate that their source/objectives partially differed somewhat, i.e., these concerned perceptive rather than knowledge-acquisitional subjects, specifically understandings of the monitoring of safe prescriptions in complex clinical cases, and of how exactly professional clinicians and pharmacists should work together collaboratively, respectively.

Additionally, to show a quite clear, albeit partial, discrimination of the post-programme responses (red) from the pre- ones (green), the three-dimensional (3D) PLS-DA scores plot shown in Figure 1 (right) also shows that the former response group was sub-clustered into three separate groups, the first co-clustering with the pre-questionnaire response group and having greater component 1 scores than the other post-programme sub-clusters. Since these post-programme (red-coloured) sub-clusters predominantly arise from their component 1 and 2 score values, those with the lowest and intermediate score indices on these reflect student response Likert scale improvement ratings, which were the highest and not so high, respectively (in this score plot, component 3 does not appear to significantly contribute towards any of these distinctions).

Moreover, the post-programme sub-cluster (red), which co-clustered with the pre-programme full cluster (green) dataset, encompasses datapoints largely reflecting the 14 student participants whose post-programme improvement score was only limited and not very dissimilar to their pre-programme values.

Similarly, MV non-parametric type EBA analysis demonstrated that all eight questions evaluated were statistically significant, with z values of 3.40, 0.95, 3.09, 2.71, 3.98, 2.94, 2.71, and 4.04 for questions 1, 2, 3, 4, 5, 6, 7, and 8, respectively (cut-off threshold value 0.946). This verified that the positive responses to each question were fully affirmed by each student participant, the greatest effect being with Q8.

### 4.3. Student Feedback and Opinions

A total of 69 out of 91 (76%) undergraduate pharmacy students between 2017–2019 completed Section 3. Out of the 112 students, only 91 answered the ‘open-ended’ questions of the questionnaire, which were subsequently analysed here. However, only 69 completed the Likert scale assessment and answered the open-ended questions (of these, 4 students abstained from completing the former evaluations). Closed question responses can be viewed in the contingency Table 3.

Following exclusion of the ‘strongly disagree’ section, which were zero for each featured cell, and participants abstaining (n = 4 for each question), these data were analysed as an χ^2^ contingency table. This test for association was statistically significant (*p* < 10^−4^), and significant contributory cells are shown in Table 3 (Fisher’s exact test). Clearly, all five of the questions placed had significantly higher positive participant fractions than the overall expected values.

Open questions were also included in the questionnaire, and these have been thematically analysed in Section 4.4.

### 4.4. Phase 2: Qualitative Studies

Responses to open-ended questions in all questionnaires and transcripts were anonymised and thematically analysed by two researchers (NL and SH), and then independently verified by two academic colleagues based at DMU.4.5. PHCT views.

Questionnaires (n = 12) were returned by two training sites. Six in-depth interviews were also conducted (4 GPs and 2 practice staff). The main themes identified were the value of the programme and its PHCT perspective. A copy of this PHCT questionnaire is available in Section S1 of the Supplementary Information.

#### 4.4.1. Value of the Programme

PHCTs and GPs strongly affirmed the positive student experience from learning in primary care. The GPs felt significant learning occurred in patient consultation skills. Through observation of GP consultations, the students learned about how to conduct a holistic interview with a patient (using the ICE (ideas, concerns, expectations) model) and how this may differ from the pharmacy consultation skills acquired through working in a hospital pharmacy setting.

GPs and practice clinical pharmacists also commented that providing feedback on the student care plan presentations (which was part of the student assessment) was particularly valuable; they felt that students had contributed meaningfully, making appropriate recommendations and, in some cases, leading to patient safety concerns requiring action (further explored in Phase 3 of the project).

The GP practice fully valued the programme, as it allowed PHCT members to develop their own understanding of the pharmacy course and profession. Indeed, it was noted that many of the GPs who participated in the teaching commented that they too had refreshed their knowledge on the science and some aspects of medicines management simply by preparing to teach the pharmacy students.

“Having the pharmacy students was a thoroughly enjoyable and engaging experience. Inter-professional education is lifelong and the opportunity to teach pharmacy students was a learning opportunity for everyone here as well.”

(Practice pharmacist)

#### 4.4.2. PHCT Perspective

PHCT staff commented on various issues that they found challenging, in particular those concerning quality assurance and logistics. All participating GP training sites had not previously accommodated pharmacy undergraduates, although they were familiar with the placement programmes for nursing and medical students, together with a range of other healthcare professionals. Comments were made on the financial incentives to support the continuation of such programmes.

Staff questioned commented on the support they received from DMU, noting that this collaboration was necessary to allow for the pedagogic principles to be aligned to the clinical teaching in primary care, and that such a partnership was required for both parties to understand the nuances of working in primary care, underpinned by the theoretical principles of pharmacy education and clinical practice. Hence, notably at the two sites, a clinical pharmacist was a key feature of the teaching strategy, as this allowed them to experience and provide viewpoints on the reality of pharmacists working in general practice, as well as the practical opportunities that students could reflect on and consider for their future career practices.

The analysis and extracts of the interviews and comments showed positive attitudes by both staff and students involved alike, which confirms learning and progression of all those involved in GPEP.

### 4.5. Students’ Views

A total of 91 out of 112 students completed the open-ended questions in Section 3, and the narratives obtained from two focus groups (n = 18) revealed two themes: the student experiences and student perspectives.

Primarily, differential data components were integrated into the research design for the qualitative analysis conducted. The free text comments, interviews, and focus group transcribed data were analysed using reflexive thematic analysis [19]. The qualitative student data scripts, interviews, and questionnaires, however, were read and explored by two of the authors (SH and NL) separately to develop an understanding of the coded and identified categories, and these viewpoints were subsequently brought together by these authors through debate and discussion, which explored the similarities and differences between participants to agree the final understandings arsing.

In the second step, however, the codes were brought together to agree a pattern of shared meaning following the six steps consisting of “Familiarisation, Coding, Generating Initial Themes, Reviewing and Developing Themes, Refining, Defining, and Naming Themes” [19]. This was conducted until “saturation” was attained. The qualitative datasets for the open-ended questions in the student and practice questionnaires were also analysed using thematic analysis techniques. This approach allowed for a deeper appreciation of the impacts of student learning against our research questions. However, since only one patient interview with a public group representative (at one practice) concerning the programme itself was completed, the investigators could only share extracts of this interview, which represents a limitation of this portion of the study.

#### 4.5.1. Student Experiences

Most students enjoyed this course, and their discussions highlighted and enabled them to consider a potential future career in primary care. Their feedback was well received during their presentations. They had gained confidence in a range of aspects of their learning, such as communication, interviewing, and consultation skills. They commented on how this increased their confidence and that the placement should have been for an extended duration.

Comparisons were made to their hospital placement programmes. Students felt welcomed during GPEP and clearly benefitted from observing other practicing healthcare professionals in a primary care environment, as supported by the quote below:

“Very interactive and beneficial for current studies and future practice! Definitely something to consider as a future career. Got a chance to discuss complex patients and put it under a pharmaceutical view. Different experience to hospital visits, where we did not get an opportunity to view patient-doctor interactions closely or even learn extensively from our fellow HCPs. Very friendly and supportive staff at Site 1.”

#### 4.5.2. Student Perspectives

Challenges were referenced. Some GP sites were located further from the DMU campus, so travel time needed consideration, and whilst reimbursement schemes were in place, unfortunately, transport was not always possible via main bus or train routes.

Whilst students appreciated the workbook material preparation and preparatory sessions, some commented on the level of clinical knowledge that was required for the programme. Those on the programme at the beginning of the academic year perceived themselves to be disadvantaged since they had yet to advance their clinical skills.

### 4.6. Patients’ Views

Five out of eight patients contacted the study organisers and consented to participate in an interview with a member of the research team. Four patients were from Site 1, and the fifth was a practice patient participation group (PPG) member from Site 2, who had also viewed some of the student presentations. Two major themes were identified: working with students and patient value.

#### Working with Students and Patient Value

Patients were notified by their GP about GPEP. They were not fully aware of when and what to expect during the consultation, leading to a sense of apprehension. They communicated that being better informed could have helped.

For the interview, most patients were pleasantly surprised by the professionalism (i.e., being made to feel comfortable and courteousness) shown by the pharmacy students and that they went beyond “asking about their medicines”. They enjoyed contributing to student learning. Some cited students as “nervous” and perhaps not being prepared enough. There was also acknowledgement that, if anything further needed to be done, it would be led by GPs or other practice colleagues. These findings are further analysed in Phase 3 of the study below.

A PPG member highlighted that person-centred care was at the heart of this programme, strongly contributing towards student learning regarding shared decision-making in primary care. This was, in this member’s view, a vital aspect of education of pharmacists in general practice and that they would be happy to participate again.

“I felt they (students) were very nervous… (did they act professionally?). Yes, I think they did. I think it was more a case of they were reticent, that was all, but no they were respectful and, you know, engaging. Once we got over the initial bit then everything went fine…(eye contact?). They did, yes.”

“The outcome (from a patient perspective) was very pleasing with no negative comments at all.”

### 4.7. Phase 3: Impact and Value

During one aspect of the assessment for academic processes (pharmaceutical care plan presentations), students recorded their findings and potential interventions. These were requested by the GPs for their healthcare assessment records and were considered by the PHCT and GPs concerned for potential interventions.

Importantly, over forty issues relating to medicine optimisations and patient safety were identified by the students. Medicine optimisation issues such as monitoring, changes in medication, deprescribing, and lifestyle changes all prompted clinical medication reviews, with some requiring immediate action. Examples of these are provided in Table 4.

Discussions with the patients concerning the optimisation of the medicines and producing a potential pharmaceutical care plan took place as a component of their learning outcomes.

As part of the academic assessment processes (presentations), students recorded their findings and potential interventions offered. These were requested by the GPs, along with healthcare assessments for their records, and were considered by the practice team and GPs for potential interventions. Patient interviews and GP interviews requesting knowledge on ‘impact’ highlighted that changes were made to patient medication records and that most of the recommendations made were actually recorded in the patient records.

Typical examples of quotes from the thematic analysis are as follows:

“The student told me the information would be given to the pharmacist, since seen the pharmacist and the GP. I [the patient], had medication reviewed and changed”

(*patient quote*).

“We have made notes of all the recommendations in the patients’ clinical record. We will prioritise all the patients seen by the students that required immediate changes, and ask the patients back for prompt review and follow up”

(*GP-pharmacist interview*).

A full summary of the qualitative analysis performed is provided in Supplementary Materials, Section S3. This features Tables entitled “Data source for the qualitative analysis” (Table S1) and “Themes identified from the analysis” (Table S2).

## 5. Discussion

This programme has demonstrated that undergraduate pharmacy placements in primary care add value to both students and practice teams. Whilst the initial aim of the programme was for the instigation of clinical placements, there appears to have been additional benefits, including a positive impact on patient care, which contributed to the clinical work of the practice. Together with undergraduate and practice staff benefits, insights have been gained into the roles and values of pharmacists in primary care.

The results from this study also identify that undergraduate student placements in primary care can positively impact practice teams’ perceptions and ***patient safety*** by enhancing medicine optimisations. Patients interviewed in the study valued their contact with students, and the interventions arising from the issues raised by the students were acknowledged by the clinicians in practice as significant improvements in patient care.

Students reported that working with GPs had improved their communication and consultation skills and allowed them to contextualise their theoretical knowledge, developments which served to increase their confidence. Evidence from both the quantitative and qualitative data from this study confirms that placements in GP practices have promoted collaborative working and learning opportunities between student pharmacists and healthcare staff within primary care settings.

### 5.1. Strengths and Limitations of the Study

Whilst the results of this study cannot be generalised, the programme achieved much local impact at the practice sites, highlighting areas of improvement in medicines adherence and optimisation. A limitation of the programme, however, was that not all undergraduate final year students at DMU could be placed in primary care in view of limited funding availability and also capacity constraints.

A further limitation was that, although the application has been approved and endorsed by internal staff Programme Management Committees, which oversee this and other educational programmes such as Interprofessional education (IPE), etc., along with our Faculty Research Ethics Committee, the absence of psychometric validations of this study survey through reliability tests also serves as a study weakness. Moreover, it was noted that only two patients consented to audio recordings during the GPEP study. Additionally, the authors advise that some caution should be taken when interpreting the results of this investigation. One further issue concerns the sample sizes of the experimental surveys performed, which might be considered limited somewhat.

It should also be acknowledged that there was a significant time lag between study data collection and publication. This relates to the researchers’ focus of work shifting during the COVID-19 pandemic; however, now that experiential placements are high up in pharmacy education, this was prioritised as an essential research approach to evaluate.

### 5.2. Comparisons of Results with Those of Related Studies Conducted

Interestingly, related UK investigations [20,21,22,23] have previously highlighted the potential benefits and barriers of partnerships between pharmacy undergraduates and members of general practice teams. As expected, the value of collaborative learning with GP academic training practices was found to be highly beneficial, and the above investigations have shown the benefits of pharmacy student placements and their learning routes in general practice. For example, one such study [20] found that this placement scheme offers an effective, attainable, and supportable teaching and educational scheme for pharmacy students, providing them with invaluable exposure to and experience of general practice environments, along with supporting work-task objectives. Moreover, a further study [21] found that this form of intervention was achievable, well received, and tolerated by patients, whilst offering a number of potential practice benefits. Although, at that stage of the work, this publication’s authors were not able to determine the cost-effectiveness of this intervention, they concluded that it provided a valuable experiential learning opportunity for pharmacy undergraduates. However, our study also drew attention to the experiences of staff and student supervisors for this programme and explored further insights into students’ education and the patients’ views, including the identification and understanding of potential medicines optimisation issues. In particular, this investigation has highlighted that the implementation of student activity described is in line with the current governmental agenda for pharmacy education, and, with suitable funding, has energised students to consider extended future roles in general practice. 

### 5.3. Implications for Research and General Practice

The General Pharmaceutical Council (GPhC) recognizes the value of such placements. At the time when the programme was developed (in 2016), initiatives of this type were in their infancy. Whilst placements have increased a little, considerable variation in the amount and type of exposure to primary care placements exists between Schools of Pharmacy at an undergraduate level [20,21,22,23], despite NHS England announcing and continuing to support national tariff funding since that time. Moreover, pharmacy undergraduate education based on evidence-based practices (as related to UK patients), with the key aims of demonstrating patient value in general practice and improving student communication/clinical skills, have yet to be tested or demonstrated [24,25].

This programme could be used as a delivery model for future pharmacy undergraduate placements. Indeed, it offers opportunities to work with local training hubs, primary care networks, and NHS England (Workforce, Training, and Development Teams) to co-create educational materials aligned to sound competencies and training standards required by academic institutions, along with the new education standards for pharmacy undergraduates advocated by the GPhC.

A common theme of interest for both the GPhC and the General Medical Council (GMC) is safe prescribing. Indeed, with an increase in patients with multiple long-term conditions with significant medicine requirements, general practitioners and clinical pharmacists must ensure regular medicine reviews, especially since these relate to patients who have complex prescription requirements [26]. This requires efficient and effective shared working practices between pharmacists and GPs. This programme demonstrates that the inclusion of the concepts early at an undergraduate level helps with the development of confidence to work in primary care [27].

## 6. Conclusions

It is essential that pharmacy undergraduates have experience in all areas of practice, particularly in primary care, where the role of the pharmacist continues to rapidly develop. The placement programme outlined herein offers undergraduates a thorough introduction to clinical work in general practice. Moreover, it is expected that the impact of this study will address political incentives for clinical pharmacy workforce development in this field and therefore closer working relationships between all PHCT members involved.

The blueprint of this programme will help aid education and training to provide a resilient and able future pharmacy workforce. This links back to key papers relating to the implementation of the five-year forward view, long-term plan, and future workforce development for pharmacists. The recent publication of the 10-Year Health Plan [28] for England also highlights potential opportunities for pharmacy teams too, enabling them to carry out wider clinical roles within their capability—rendering excellent training at an undergraduate level, as evidenced in this programme.

## Figures and Tables

**Table 1:** General Practice Pharmacy Education Programme (GPEP).

Pharmacy Undergraduate Educational Programme	
**Week 0 (0.5 day)**	Preparation of student materials and GP governance processes.
Week 1 (0.5 day)	Orientation to GP practice with clinical pharmacists and/or GP trainers; Introduction to GP practice systems.
Week 2 (0.5 day)	Case-based learning; Medicine reconciliation; Feedback from Practice Trainers.
Week 3 (0.5 day)	Consultation skills—observation of patient consultations; Medication reviews; Feedback from Practice Trainers.
Week 4 (0.5 day)	Interviewing the patient; Feedback from Practice Trainers.
Week 5 (0.5 day)	Presentation of a patient pharmaceutical care plan; Assessment by GP trainers and DMU academic staff.

**Table 2:** Results from student self-rating ability on a five-point Likert scale.

Question Code	Learning Objective	Pre- (Mean)	Post- (Mean)	*p* Values
Q1	Demonstrate what is meant by evidence-based medicine and its implications on safe prescribing	3.0	4.2	<10^−4^
Q2	Understand pharmacology of the drugs used in the care of complex patients	2.9	4.1	<10^−4^
Q3	Understand the rationale underpinning medicines optimization	2.9	4.1	<10^−4^
Q4	Check if drugs are safely prescribed	3.2	4.2	<10^−4^
Q5	Understand if current prescribing encompasses safe prescribing practice	2.7	4.0	<10^−4^
Q6	Analyse how professionals from medicine and pharmacy should work together	3.2	4.4	<10^−4^
Q7	Demonstrate an understanding of patient care through listening to members of the multidisciplinary team	3.2	4.4	<10^−4^
Q8	Construct a holistic plan of patient care after interviewing a patient (and their family/carers) and reviewing their case	2.6	4.2	<10^−4^

**Table 3:** Contingency table results of student responses (n = 69 participants). Key: *, **, and *** indicate statistical significance *p* values for the χ^2^ statistic at the <0.05, <0.01, and <0.0001 levels, respectively. Four student respondents abstained from answering the Likert scale questionnaire assessment.

	Number of Responses (%)					
Questions	Strongly Disagree	Disagree	Neither Agree nor Disagree	Agree	Strongly Agree	Abstained
I have achieved the learning outcomes during my placement	0 (0%)	3 (4%) ***	2 (3%)	36 (52%) **	24 (35%) *	4 (6%)
I have received enough relevant information for my placement	0 (0%)	0 (0%)	4 (6%)	35 (50%)	26 (38%) *	4 (6%)
I have enjoyed the placements	0 (0%)	0 (0%) ***	1 (1%) *	24 (35%)	40 (58%) *	4 (6%)
The placement was well organised	0 (0%)	6 (9%) *	9 (13%) *	21 (30%)	29 (42%)	4 (6%)
The placement was beneficial to my role as a pharmacist	0 (0%)	1 (1%)	4 (6%)	19 (28%) *	41 (59%) *	4 (6%)

**Table 4:** Patient safety and medicines optimisation impact.

Issues Raised	Frequency	Examples of Interventions by the Practice Teams
Immediate action to change medication	3	Review of frequency of opiate prescribing; Change DOAC (Direct Oral Anticoagulant); Optimisation of statin treatment.
Changes in medication	22	Change to a more suitable DOAC; Increase dose of Amlodipine to reduce blood pressure; Increase Bisoprolol dose to reduce blood pressure; Start Atorvastatin for primary prevention; Increase Ramipril dose to manage blood pressure; Switch Calcichew to D3 Forte; Consider Montelukast for seasonal asthma; Aspirin reduced to a dose of 75 mg daily.
Identification of important side-effects	1	Review statin choice in view of myalgia.
Deprescribing	6	Terminate inhalers since they were no longer required; Stop repeated hydrocortisone prescription, since it was not ordered for four years; Trial without Atorvastatin.
Clinical monitoring	5	Recommend increased potassium monitoring; Recommend monitoring of anti-diabetic treatments; Reduce HCA blood pressure checks from 4 monthly to annually.
Referral to specialist	1	Patient requested to switch from warfarin to DOAC. This prompted a clinician review of treatment and a subsequent referral—when previously discharged from a haematology clinic 7 years ago, they were requested to have 3-yearly specialist clinical reviews of anticoagulation treatment.
Recommendation of over-the-counter medication	1	Commence Paracetamol 500 mg 1–2 qds prn treatment for osteoarthritis.
Changes in lifestyle (including social prescribing)	6	Increase dietary intake of healthy foods (fruits, vegetables, etc.); Consider weight loss programme; Start exercising.

## Data Availability

Data presented in this study are available through contact with the corresponding author of this article (email: amit.bharkhada@dmu.ac.uk).

## Supplementary Materials

The following supporting information can be downloaded at: https://www.mdpi.com/article/10.3390/clinpract16010005/s1, Table S1.1: Pre-Placement Questions; Table S1.2: Post-Course Questions; Table S1.3: Feedback Indication; Table S3.1. Data source for the qualitative analysis; Table S3.2: Themes identified from the analysis [29]; Figure S2.1. Two-dimensional (2D) PCA PC2 versus PC1 scores plots for the analysis of this study’s Likert scale data, using (a) Polychoric, (b) Spearman Rank and (c) Pearson correlation coefficients within the correlation matrix for analysis. Corresponding PC3 versus PC1 plots using Polychoric, Spearman Rank and Pearson correlation coefficients are shown in (d), (e) and (f) respectively. Label 0 and Label 1 observations are indicated as green or blue circles, respectively. PCA cluster centroid score values for questionnaire surveys conducted pre- and post-programme (black circles) were found to be −1.81 and +1.81 (Polychoric), −1.80 and +1.80 (Spearman’s Rank) and −1.80 and +1.80 (Pearson) for PC1 in groups 1 and 0, respectively; +0.04 and −0.04 (Polychoric), −0.16 and +0.16 (Spearman’s Rank) and +0.07 and −0.07 (Pearson) for PC2 in groups 1 and 0, respectively; and −0.14 and +0.14 (Polychoric), 0.10 and −0.10 (Spearman’s Rank) and −0.18 and +0.18 (Pearson) for PC3 in groups 1 and 0, respectively. Percentages of the total variance captured for plots (a), (b), (c), (d), (e) and (f) were 83.4, 77.8, 76.6, 82.4, 77.4 and 76.4%, respectively.
