# Supplementary material for: Pharmacy Undergraduate Education: Can Student Primary Care Placements Add Value to Learning and Teaching?
Source: Clin Pract. 2025 Dec 25;16(1):5. doi: 10.3390/clinpract16010005 (PMC12839822; doi:10.3390/clinpract16010005)
Supplement: Supplementary file 1 [file clinpract-16-00005-s001.zip › clinpract-3956428-supplementary.pdf]

Pharmacy Undergraduate Education: Can Student Primary Care Placements Add Value to Learning and Teaching?

Amit Bharkhada [1,2]*, Neena Lakhani [1], Sandra Hall [1] and Martin Grootveld [1]

[1]Leicester School of Pharmacy, De Montfort University (DMU), The Gateway, Leicester, LE1 9BH, UK;
[2]Leicester, Leicestershire, Rutland (LLR) Training Hub, Leicester, LE72EQ, UK
* Correspondence: amit.bharkhada@dmu.ac.uk
Author contact email: amit.bharkhada@dmu.ac.uk

SUPPLEMENTARY INFORMATION SECTION S1

Student Clinical Placement Evaluation Questionnaires

DE MONTFORT UNIVERSITY LEICESTER

Clinical Placements

Phar 4601

Personal details

Gender: **MALE** ☐ **FEMALE** ☐

PLEASE TICK ✔

Table S1.1: Pre-Placement Questions.

Please tick which level of knowledge best describes your abilities before you start on this course. Score: 1. little understanding to (5) a great deal of understanding using a √.

Intended Learning Outcomes	1	2	3	4	5
**My ability to demonstrate what is meant by evidence-based medicine and its implications on safe prescribing**					
**My ability to understand pharmacology of the drugs used in the care of complex patients**					
**My ability to understand the rationale underpinning medicines optimisation**					
**My ability to check if drugs are safely prescribed**					
**My ability to understand if current prescribing encompasses safe prescribing practice**					
**My ability to analyse how professionals from medicine and pharmacy should work together.**					
**My ability to demonstrate an understanding of patient care through listening to members of the multidisciplinary team**					
**My ability to construct a holistic plan of patient care after interviewing a patient (and their family/carers) and reviewing their case**					

Table S1.2: Post-Course Questions.

Please tick which level of knowledge best describes your abilities following this short course. Scheme 1. little understanding to (5) a great deal of understanding.

Intended Learning Outcomes	1	2	3	4	5
My ability to demonstrate what is meant by evidence-based medicine and its implications on safe prescribing					
My ability to understand pharmacology of the drugs used in the care of complex patients					
My ability to understand the rationale underpinning medicines optimisation					
My ability to check if drugs are safely prescribed					
My ability to understand if current prescribing encompasses safe prescribing practice					
My ability to analyse how professionals from medicine and pharmacy should work together.					
My ability to demonstrate an understanding of patient care through listening to members of the multidisciplinary team					
My ability to construct a holistic plan of patient care after interviewing a patient (and their family/carers) and reviewing their case					

Table S1.3: Feedback Indication.

Please indicate your feedback for the placement (tick relevant box below and add comments if required). 1 – strongly disagree, 2 – disagree, 3 – neither agree or disagree, 4- agree, 5- strongly agree.

	**1**	**2**	**3**	**4**	**5**
**I have achieved the learning outcomes during my placement.**					
**Further comments:**					
**I received enough relevant information for my placement.**					
**Further comments:**					
**I enjoyed the placement.**					
**Further comments:**					
**The placement was well organised.**					
**Further comments:**					
**The placement has been beneficial to my role as a pharmacist.**					
**Further comments:**					

Thank you.

Supplementary Material Section S2

Principal Component Analysis of Experimental Study Likert Scale Data: Correlation Coefficient Class Dependence

Principal Component Analysis (PCA) of Experimental Study Likert Scale Data

(a)

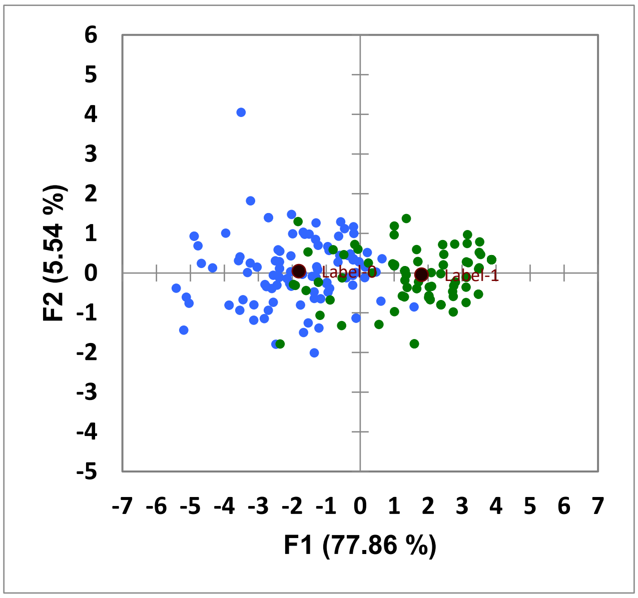

(b)

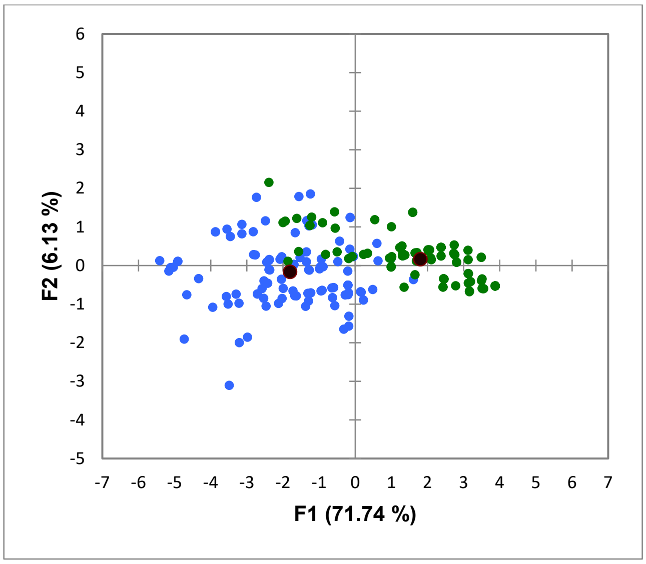

(c)

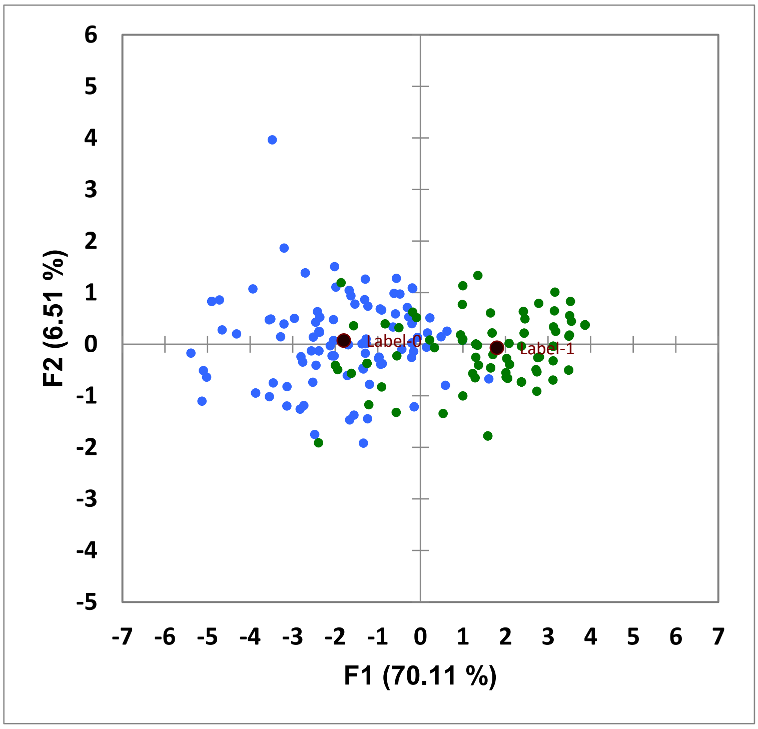

(d)

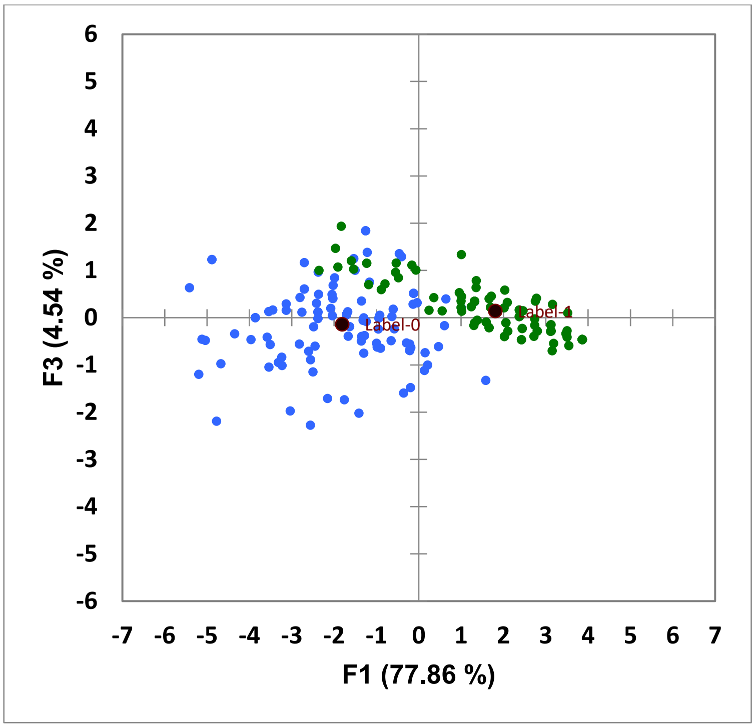
F3 (4.54 %)
F1 (77.86 %)
Label-0
Label-1
6 5 4 3 2 1 0 -1 -2 -3 -4 -5 -6
-7 -6 -5 -4 -3 -2 -1 0 1 2 3 4 5 6 7

(e)

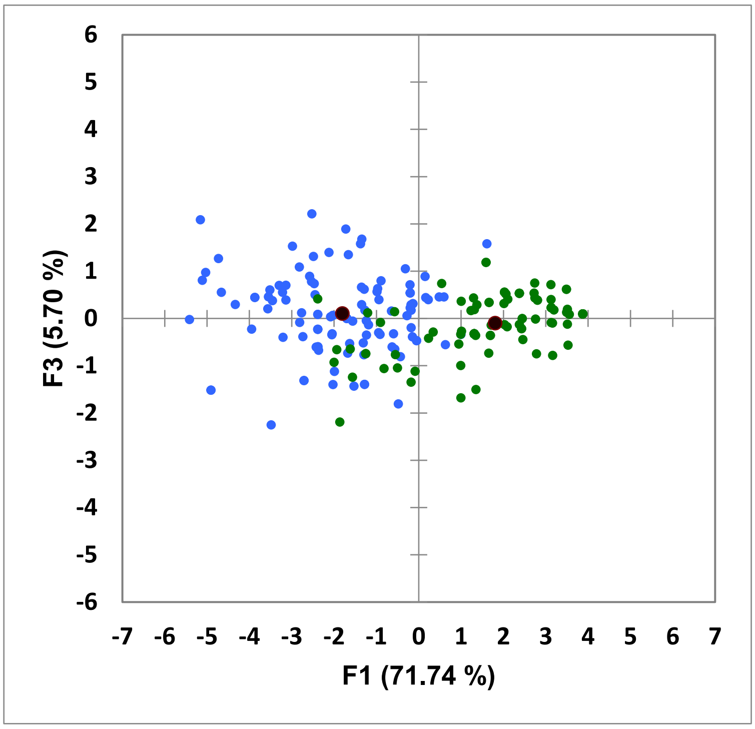
F3 (5.70 %)
F1 (71.74 %)
6 5 4 3 2 1 0 -1 -2 -3 -4 -5 -6
-7 -6 -5 -4 -3 -2 -1 0 1 2 3 4 5 6 7

(f)

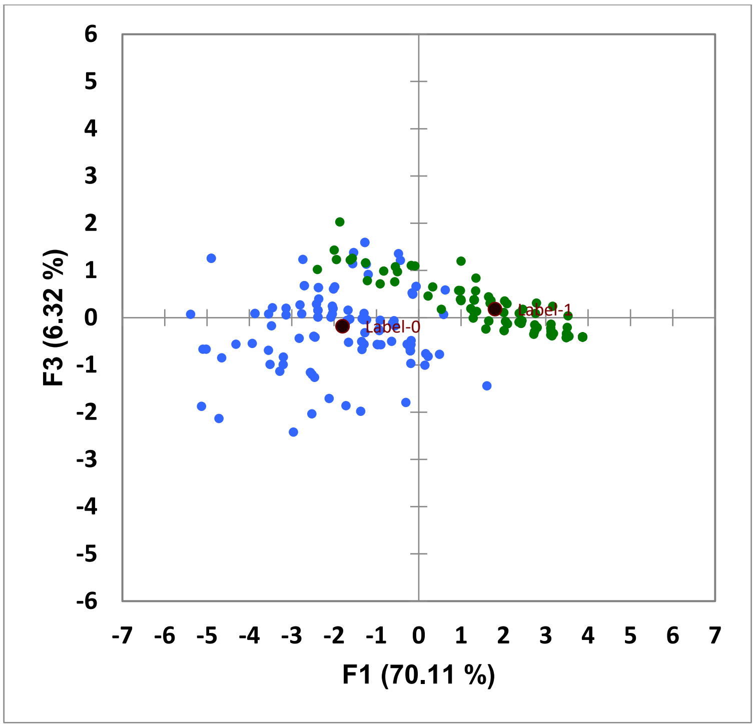

Figure S2.1. Two-dimensional (2D) PCA PC2 *versus* PC1 scores plots for the analysis of this study's Likert scale data, using (a) Polychoric, (b) Spearman Rank and (c) Pearson correlation coefficients within the correlation matrix for analysis. Corresponding PC3 *versus* PC1 plots using Polychoric, Spearman Rank and Pearson correlation coefficients are shown in (d), (e) and (f) respectively. Label 0 and Label 1 observations are indicated as green or blue circles, respectively. PCA cluster centroid score values for questionnaire surveys conducted pre- and post-programme (black circles) were found to be -1.81 and +1.81 (Polychoric), -1.80 and +1.80 (Spearman's Rank) and -1.80 and +1.80 (Pearson) for PC1 in groups 1 and 0, respectively; +0.04 and -0.04 (Polychoric), -0.16 and +0.16 (Spearman's Rank) and +0.07 and -0.07 (Pearson) for PC2 in groups 1 and 0, respectively; and -0.14 and +0.14 (Polychoric), 0.10 and -0.10 (Spearman's Rank) and -0.18 and +0.18 (Pearson) for PC3 in groups 1 and 0, respectively. Percentages of the total variance captured for plots (a), (b), (c), (d), (e) and (f) were 83.4, 77.8, 76.6, 82.4, 77.4 and 76.4%, respectively.

Supplementary Material: Section S3

Qualitative Analysis Summary/Themes Identified from the Analysis

Qualitative analysis summary: Phase 2.

Table S3.1: Data source for the qualitative analysis.

	**Questionnaires (open and closed)**	**Focus groups**	**One-to-one interviews**	**Field notes**
**Students**	**91/113 (80%) completed open-ended questions**	**2 (n = 18)**<br>10 (Loughborough)<br>8 (Syston)	n/a	n/a

**Practice staff (including GPs)**	**12/14 questionnaires returned (86%) from 2 sites**<br>Site 1: 5 GPs, 1 IT administrator, 1 practice manager.<br>Site 2: 2 GPs and 3 Practice managers	n/a	6 patients,<br>4 GPs,<br>2 practice staff	One PPI Rep. (Syston);<br>One PPI Rep, (Saffron Lane);<br>One Practice Pharmacist<br>(Corby).
**Patients**	0	0	6	0

Abbreviation: n/a, not applicable.

Questionnaire: Section 3 of the questionnaire
Focus group and semi-structured interviews: Topic guides were employed
Thematic analysis (i.e., the Braun and Clarke approach [19]) was used for the analysis. This strategy represents a six-phase qualitative research protocol for the identification and interpretation of dataset patters, such as those derived from interviews or focus groups [19]. Limitation for field notes: field notes were obtained via email or anecdotal telephone conversations.

Results
Table S3.2: Themes identified from the analysis

**Main themes**	**Categories**
Student views (open- ended questions and focus groups)	Preparation; Expectations; Enjoyment; Value of the programme; Course improvement; Learning and reflection; Impact on future career path
Stakeholders' views (semi structured interviews)	Practice preparation; Value to the practice; Student enjoyment<br>Course development; Collaboration; Views on Student experience;<br>Impact
Service users' views (patients)	Professionalism; Communication; Medicines use;<br>Engagement with the programme

Full original quotes are available from the manuscript authors.

Reference
[19] Braun V., Clarke V. (2006). Using thematic analysis in psychology. *Qualitative Research in Psychology*, 3(2), 77–101. https://doi.org/10.1191/1478088706qp063oa
